# The Role of Ultrasonography and Magnetic Resonance Imaging in the Evaluation of Adnexal Masses With Histopathological Correlation

**DOI:** 10.7759/cureus.96259

**Published:** 2025-11-06

**Authors:** Chethana S Reddy, A Baskar, M Kalaichezhian, G Murugan

**Affiliations:** 1 Radiology, Sree Balaji Medical College and Hospital, Chennai, IND

**Keywords:** adnexal masses, histopathological diagnosis, magnetic resonance imaging (mri), malignancy, ultrasonography

## Abstract

Background: Accurate evaluation of adnexal masses is crucial for appropriate management, distinguishing benign from malignant lesions, and guiding surgical planning. Ultrasonography (USG) is commonly used as a first-line modality, while magnetic resonance imaging (MRI) provides detailed tissue characterization. This study aimed to compare the diagnostic performance of USG and MRI in the assessment of adnexal masses and to correlate imaging findings with histopathological examination (HPE).

Methods: A prospective study was conducted on 67 patients presenting with adnexal masses. All patients underwent USG and MRI evaluation, including assessment of tumor size, surface, septations, solid components, vascularity, ascites, and lymphadenopathy. Imaging findings were correlated with HPE results. Diagnostic performance metrics, such as sensitivity, specificity, positive predictive value (PPV), negative predictive value (NPV), and accuracy, were calculated for each modality.

Results: USG identified 76.1% of masses as benign and 23.9% as malignant, while MRI identified 79.1% as benign and 20.9% as malignant. MRI demonstrated higher sensitivity (98.2% vs 92.6%), specificity (100% vs 92.3%), PPV (100% vs 98.0%), NPV (92.9% vs 75.0%), and overall diagnostic accuracy (98.5% vs 92.5%) compared with USG. MRI also better detected septations, solid components, ascites, and lymphadenopathy. Histopathology confirmed that benign cystic lesions were the most common pathology, with a small proportion of malignant tumors. The correlation between USG and MRI findings showed high agreement, indicating the reliability of USG as an initial assessment tool.

Conclusion: USG is a dependable first-line imaging modality for evaluating adnexal masses, while MRI provides superior diagnostic confidence, particularly in complex or suspicious cases. Combined imaging and histopathological correlation optimize diagnostic accuracy, guide surgical planning, and improve patient outcomes.

## Introduction

Adnexa refers to the anatomical region of the female pelvis that contains the ovaries, fallopian tubes, round ligament, and other associated structures originating from related embryologic tissues. The adnexal region is of critical clinical importance because it harbors several structures prone to pathological changes. Among these, adnexal masses are the most prevalent pathological findings encountered in women during routine gynecologic evaluation or imaging investigations [1]. These masses encompass a wide spectrum of etiologies, ranging from functional cysts and benign neoplasms to malignant tumors, and therefore represent a significant diagnostic challenge for clinicians and radiologists alike.

One of the most crucial steps in the management of adnexal masses is distinguishing benign from malignant tumors [2]. Accurate preoperative characterization of these masses significantly influences patient care, therapeutic planning, and surgical decision-making. In particular, for young women of reproductive age, determining the nature of an adnexal mass before surgery is especially important to preserve future fertility and avoid unnecessary radical procedures. Misclassification may lead either to overtreatment with extensive surgery in benign conditions or to undertreatment in cases of malignancy. Hence, accurate diagnostic assessment is paramount.

Imaging plays a central role in the initial detection and further evaluation of adnexal lesions. The two principal imaging modalities used are ultrasonography (USG) and magnetic resonance imaging (MRI) [3]. Ultrasound is widely available, cost-effective, and noninvasive, making it the first-line investigation in most cases. It offers real-time imaging and allows assessment of the size, shape, location, and vascularity of adnexal masses. However, despite these advantages, USG has several limitations, including a restricted field of view, operator dependency, and possible obscuration of the adnexa by bowel gas [4]. Furthermore, complex or indeterminate masses on ultrasound often require additional imaging for better tissue characterization.

MRI has emerged as a valuable complementary tool in the evaluation of adnexal masses. With its superior soft tissue resolution, excellent contrast differentiation, and multiplanar imaging capability, MRI enables a more accurate and detailed assessment of adnexal pathology [5]. MRI can delineate the internal architecture, composition, and extent of lesions with high precision, thereby providing crucial information that can guide appropriate clinical management. In particular, MRI is advantageous in distinguishing solid from cystic components, detecting hemorrhage, fat, or fibrous tissue, and identifying local invasion or peritoneal spread, all of which are important in differentiating benign from malignant lesions.

Therefore, determining the diagnostic performance of these imaging modalities is essential. Several studies have emphasized the importance of correlating imaging findings with histopathology, which remains the gold standard for confirming the nature of adnexal lesions [6]. Imaging not only aids in detecting these masses but also contributes significantly to understanding their origin and biological behavior [7]. Accurate characterization of adnexal lesions can influence the therapeutic pathway: less invasive surgery may be appropriate for lesions suspected to be benign, whereas extensive staging surgery is warranted for lesions highly suspicious for malignancy. Hence, reliable preoperative characterization helps in selecting the optimal surgical approach and improves patient outcomes.

In current clinical practice, USG and MRI are considered complementary rather than competing modalities in the evaluation of adnexal masses [8]. USG typically serves as the initial screening tool because of its accessibility and ease of use, whereas MRI is often employed for further characterization of indeterminate masses detected on ultrasound and for detailed surgical planning [9,10]. Nonetheless, final confirmation of the nature of these lesions relies on histopathological examination. A comprehensive evaluation incorporating both USG and MRI findings, followed by histopathological correlation, thus ensures accurate diagnosis and appropriate management of adnexal masses.

This study was conducted to evaluate the role of ultrasonography and magnetic resonance imaging in the assessment of adnexal masses, with correlation to histopathological findings. The objective was to detect and characterize adnexal mass lesions using USG and MRI with respect to their size, shape, location, nature, extent, borders, content, and vascularity, and to assess the relative role of USG and MRI in evaluating adnexal masses while correlating their findings with the gold standard test (histopathological examination) wherever possible.

## Materials and methods

Study design and setting

This hospital-based cross-sectional study was conducted in the Department of Radiodiagnosis and Imaging at Sree Balaji Medical College and Hospital, Chennai, Tamil Nadu, India, over a period of 18 months from July 2023 to January 2025. The study was self-funded, and a purposive sampling method was adopted to recruit participants. Women presenting with clinical suspicion of adnexal masses and referred from the Department of Obstetrics and Gynecology to the Department of Radiodiagnosis for imaging formed the study population. Patients who fulfilled the eligibility criteria and provided informed written consent were consecutively enrolled until the required sample size was achieved.

All enrolled patients underwent imaging evaluation at the Department of Radiodiagnosis. The study protocol included initial assessment with transabdominal sonography (TAS) and transvaginal sonography (TVS), followed by MRI of the pelvis using a 3 Tesla scanner with abdominal surface coils. Contrast-enhanced MRI was performed whenever indicated based on the nature of the lesion and clinical suspicion. After imaging, the patients were followed up to obtain surgical and histopathological reports whenever operative intervention was done, in order to correlate imaging findings with the final histopathological diagnosis. This setting allowed for a structured and sequential evaluation of adnexal masses, beginning with initial screening using ultrasound, followed by confirmatory and detailed characterization with MRI, and ultimately comparison with the gold standard histopathological examination.

Study population

The study included female patients aged above 18 years who were clinically suspected to have adnexal mass lesions. These patients were referred from the Department of Obstetrics and Gynecology with symptoms such as pelvic pain, irregular menstruation, or abnormal vaginal bleeding suggestive of adnexal pathology. Patients in whom ultrasound of the abdomen and pelvis yielded inconclusive findings and those who were scheduled for surgery and underwent MRI as part of preoperative evaluation were also included. Patients were excluded if they had contraindications to MRI, such as cochlear implants, older-generation cardiac pacemakers, aneurysm clips, or other noncompatible metallic implants; if they had a history of claustrophobia or intolerance to MRI examination; or if they declined to provide informed consent to participate in the study.

Sample size

The sample size was calculated based on the expected prevalence of adnexal masses in the population, the level of precision desired, and the confidence interval chosen. Considering a 7% expected prevalence, an allowable error of 5%, and a 95% confidence level, the required sample size was estimated to be 67. Hence, the final sample consisted of 67 women who fulfilled the inclusion criteria.

Operational definitions

For the purpose of this study, MRI was defined as an advanced cross-sectional imaging modality that uses strong magnetic fields and radiofrequency pulses to produce detailed multiplanar images of internal body structures. MRI works by detecting signals from hydrogen nuclei in tissues, which are then processed to form high-resolution images, providing excellent soft tissue contrast and allowing accurate characterization of adnexal lesions. USG was defined as a real-time imaging technique utilizing high-frequency sound waves to visualize the internal organs and tissues of the body. It is a noninvasive, radiation-free modality widely used as a first-line investigation for evaluating pelvic and adnexal pathologies. Histopathology was defined as the microscopic examination of tissue samples obtained through biopsy or surgical excision, following processing, paraffin embedding, sectioning, and staining, most commonly with hematoxylin and eosin, and is considered the gold standard for definitive diagnosis and characterization of adnexal lesions.

Imaging protocol

All patients underwent TAS using a curvilinear probe with a frequency of 2-5 MHz and TVS using an endocavity probe with a frequency of 6-7 MHz on a LOGIQ P7 ultrasound machine (GE HealthCare, Chicago, Illinois). MRI of the pelvis was performed using a SIGNA Pioneer 3.0 Tesla scanner with an abdominal surface coil. The standard protocol included T1-weighted and T2-weighted sequences in axial, sagittal, and coronal planes; fat-suppressed T2-weighted images; diffusion-weighted imaging (DWI); and dynamic contrast-enhanced and postcontrast T1-weighted images whenever needed. These comprehensive imaging protocols ensured accurate assessment of adnexal lesions with respect to their morphology, internal architecture, and tissue composition.

Clinical assessment and imaging evaluation

Each participant underwent detailed clinical assessment, including documentation of demographic and clinical data such as name, age, hospital identification number, parity, comorbidities, presenting symptoms, and prior imaging or operative records. All patients were first evaluated using transabdominal and transvaginal ultrasound examinations. During ultrasound, the size, shape, location, and laterality of the lesions were assessed, along with their internal echotexture (solid, cystic, or mixed); presence of septations or papillary projections; wall thickness; calcifications; hemorrhage; and vascularity using color Doppler. The presence of ascites, peritoneal deposits, or other associated pelvic abnormalities was also documented [11].

Patients with indeterminate or suspicious findings on ultrasound or those planned for surgery underwent MRI of the pelvis. MRI images were evaluated for lesion morphology, signal characteristics, internal composition such as fat, hemorrhage, fibrous tissue, or solid components, and enhancement patterns after contrast administration. The margins of the lesions, local invasion into surrounding structures, and the presence of associated features such as pelvic lymphadenopathy, ascites, or peritoneal implants suggestive of malignancy were also recorded. MRI provided additional information to classify lesions more confidently as benign or malignant, to delineate their exact extent, and to assist in surgical planning [12].

Histopathological correlation

Whenever patients underwent surgical excision of the adnexal masses, their histopathology reports were retrieved from the Medical Records Department. The imaging findings from ultrasound and MRI were then compared with histopathological diagnoses, which served as the gold standard reference for classification of the lesions as benign or malignant. This correlation helped assess the diagnostic accuracy and reliability of the two imaging modalities.

Ethical considerations

This study was conducted in compliance with the ethical principles outlined in the Declaration of Helsinki. Approval for the study protocol was obtained from the Institutional Ethics Committee of Sree Balaji Medical College and Hospital before commencing data collection. Informed written consent was obtained from all participants after explaining the purpose, procedures, potential benefits, and minimal risks associated with the study in a language they could understand. Participation was entirely voluntary, and patients had the right to withdraw from the study at any stage without affecting their routine clinical care. Confidentiality and anonymity of patient information were strictly maintained throughout the study. No personal identifiers were included in the dataset used for analysis, and all patient details were coded and stored securely. Because MRI examinations were performed only when clinically indicated or as part of preoperative surgical planning, no additional financial burden or risk was imposed on the participants. There was no anticipated physical, psychological, social, or legal harm from participation.

Statistical analysis

All data collected were entered into Microsoft Excel spreadsheets (Microsoft Corporation, Redmond, Washington) and later analyzed using IBM SPSS Statistics for Windows, Version 21 (Released 2012; IBM Corp., Armonk, New York) and OpenEpi version 2.3.1 (OpenEpi, Atlanta, Georgia). Descriptive statistics such as mean and standard deviation were used to summarize continuous variables like age, while frequencies and percentages were used for categorical variables such as lesion type. The diagnostic accuracy of ultrasound and MRI was determined using sensitivity, specificity, positive predictive value, negative predictive value, and overall accuracy, considering histopathology as the reference standard. Comparative analysis of categorical variables between imaging modalities and histopathological outcomes was performed using the chi-square test or Fisher’s exact test as appropriate, and continuous variables were compared using the independent t-test. A p-value less than 0.05 was considered statistically significant for all analyses.

## Results

Basic characteristics

The study population included women aged between 30 and over 60 years, with the majority of participants in the 50-60-year age group (24, 35.8%), followed closely by those aged 30-40 years (23, 34.3%), more than 60 years (14, 20.9%), and 40-50 years (6, 9.0%). Regarding clinical presentation, lower abdominal pain was the most common symptom, reported by 61 participants (91%), followed by pelvic heaviness in 30 patients (44.8%), urinary complaints in 12 patients (17.9%), and the presence of an abdominal mass in only one patient (1.5%). In terms of obstetric history, most women had parity between one and three (43, 64.2%), while 23 participants (34.3%) had parity greater than three, and only one woman (1.5%) was nulliparous. A previous cesarean section was reported in 15 participants (22.4%), whereas 52 women (77.6%) had no prior cesarean delivery. Regarding menopausal status, 28 participants (41.8%) were postmenopausal, and 39 women (58.2%) were premenopausal (Table 1).

**Table 1:** Demographic and clinical characteristic of study participants (n=67) This table shows the demographic (age) and clinical characteristics (obstetric, menopausal, and c-section details) of study participants.

Parameter	Category	Frequency	Percentage (%)
Age (years)	30 to 40	23	34.3
	40 to 50	6	9.0
	50 to 60	24	35.8
	>60	14	20.9
Clinical presentation	Pelvic heaviness	30	44.8
	Lower abdominal pain	61	91
	Urinary complaints	12	17.9
	Abdominal mass	1	1.5
Obstetric history	Nullipara	1	1.5
	Parity 1 to 3	43	64.2
	Parity >3	23	34.3
Previous C-section	Yes	15	22.4
	No	52	77.6
Menopause status	Yes (postmenopausal)	28	41.8
	No (premenopausal)	39	58.2

USG findings of the tumor

On USG, the majority of adnexal masses were characterized as benign, with 51 cases (76.1%), while 16 masses (23.9%) were reported as malignant. Regarding tumor size, 15 masses (22.4%) measured less than 5 cm, 32 masses (47.8%) measured between 5 and 10 cm, 17 masses (25.4%) ranged from 10 to 15 cm, and only 3 masses (4.5%) exceeded 15 cm in diameter. The tumor surface was regular in 41 cases (61.2%) and irregular in 26 cases (38.8%). Septations were present in 23 masses (34.3%) and absent in 44 masses (65.7%); among the 23 masses with septations, 16 (69.6%) had septal thickness less than 3 mm, whereas 7 (30.4%) had septa thicker than 3 mm. Ascites was noted in 9 patients (13.5%), while it was absent in 58 patients (86.5%). Solid components were observed in 23 masses (34.3%), with the remaining 44 masses (65.7%) lacking solid areas. On color Doppler evaluation, increased vascularity was detected in 22 masses (32.8%), whereas 45 masses (67.2%) demonstrated normal vascularity (Table 2).

**Table 2:** Distribution of USG findings of the adnexal mass among the study participants (n = 67) This table shows the distribution of USG findings of the adnexal mass among the study participants classified based on tumor size, surface, septal thickness, vascularity, and components. USG: ultrasonography.

Parameter	Category	Frequency	Percentage (%)
Scan findings	Benign	51	76.1
	Malignant	16	23.9
Tumor size	<5 cm	15	22.4
	5–10 cm	32	47.8
	10–15 cm	17	25.4
	>15 cm	3	4.5
Tumor surface	Regular	41	61.2
	Irregular	26	38.8
Septations	Present	23	34.3
	Absent	44	65.7
Septal thickness (n = 23)*	<3 mm	16	69.6*
	>3 mm	7	30.4*
Ascites	Present	9	13.5
	Absent	58	86.5
Solid components	Present	23	34.3
	Absent	44	65.7
Vascularity (Doppler)	Increased	22	32.8
	Normal	45	67.2

Table 3 shows the findings on MRI. A total of 53 adnexal masses (79.1%) were classified as benign, while 14 masses (20.9%) were characterized as malignant. Tumor size varied, with 10 masses (14.9%) measuring less than 5 cm, 36 masses (53.7%) measuring between 5 and 10 cm, 15 masses (22.4%) ranging from 10 to 15 cm, and 6 masses (9.0%) exceeding 15 cm in diameter. Regarding tumor surface, 38 masses (56.7%) exhibited a regular surface, whereas 29 masses (43.3%) had an irregular surface. Septations were observed in 32 masses (47.8%) and were absent in 35 masses (52.2%); within the subset of masses with septations, 13 (40.6%) had septal thickness less than 3 mm, and 19 (59.4%) had septal thickness greater than 3 mm. Ascites was present in 10 patients (15.0%) and absent in 57 patients (85.0%), while lymphadenopathy was noted in 4 patients (6.0%) and absent in 63 patients (94.0%). Solid components were detected in 32 masses (47.8%), with the remaining 35 masses (52.2%) lacking solid areas.

**Table 3:** Distribution of MRI findings of the adnexal mass among the study participants (n = 67) *Percentages for septal thickness are within the subset of cases where septations were assessed.

Parameter	Category	Frequency	Percentage (%)
Scan findings	Benign	53	79.1
	Malignant	14	20.9
Tumor size	<5 cm	10	14.9
	5–10 cm	36	53.7
	10–15 cm	15	22.4
	>15 cm	6	9.0
Tumor surface	Regular	38	56.7
	Irregular	29	43.3
Septations	Present	32	47.8
	Absent	35	52.2
Septal thickness (n = 32)*	<3 mm	13	40.6*
	>3 mm	19	59.4*
Ascites	Present	10	15.0
	Absent	57	85.0
Lymphadenopathy	Present	4	6.0
	Absent	63	94.0
Solid components	Present	32	47.8
	Absent	35	52.2

**Figure 1:**
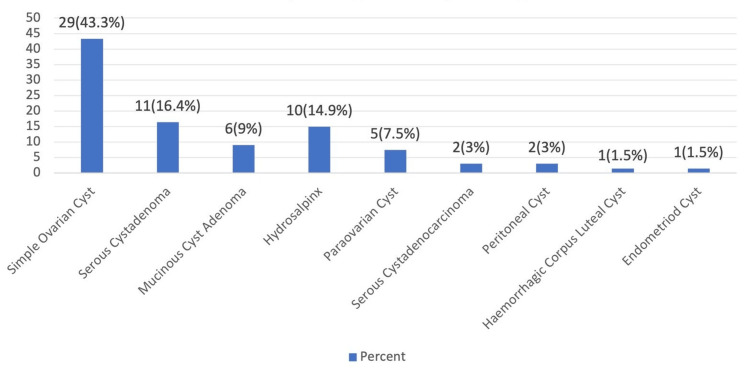
Distribution depending on histopathological examination (n = 67) This figure shows the findings of histopathological examination of adnexal masses, classified as simple ovarian cysts, accounting for 29 cases (43.3%). Serous cystadenomas were observed in 11 patients (16.4%), while mucinous cystadenomas were reported in 6 patients (9.0%).

Figure 1 shows the findings of histopathological examination. The majority of adnexal masses were simple ovarian cysts, accounting for 29 cases (43.3%). Serous cystadenomas were observed in 11 patients (16.4%), while mucinous cystadenomas were reported in 6 patients (9.0%). Hydrosalpinx was identified in 10 patients (14.9%), and paraovarian cysts were noted in 5 patients (7.5%). Malignant lesions included serous cystadenocarcinoma, seen in 2 patients (3.0%). Other less common findings included peritoneal cysts in 2 patients (3.0%), a hemorrhagic corpus luteal cyst in 1 patient (1.5%), and an endometrioid cyst in 1 patient (1.5%).

Table 4 shows the comparison of USG and MRI findings. The majority of adnexal masses were benign on both modalities, with 51 (76.1%) on USG and 53 (79.1%) on MRI, while a smaller proportion were malignant, 16 (23.9%) on USG versus 14 (20.9%) on MRI, showing no statistically significant difference (p = 0.33). Tumor size distribution was similar between the two modalities, with no significant difference. Tumor surfaces were predominantly regular on USG (41, 61.2%), whereas MRI detected slightly more irregular surfaces (29, 43.3%), which was not statistically significant (p = 0.29). Septations were more frequently identified on MRI (32, 47.8%) than on USG (23, 34.3%), showing borderline significance (p = 0.05). Solid components were also noted more often on MRI (32, 47.8%) compared with USG (23, 34.3%), again with borderline significance (p = 0.05). Ascites was absent in the majority of cases on both USG and MRI, and lymphadenopathy was detected in a few cases on MRI (4, 6%). Color Doppler evaluation on USG showed increased vascularity in 22 (32.8%) masses and normal vascularity in 45 (67.2%), while this parameter was not assessed on MRI. Overall, MRI showed slightly higher detection of septations and solid components, but most other findings were comparable between the two imaging modalities.

**Table 4:** Comparison of USG and MRI findings of the adnexal mass among the study participants (n = 67) *Chi-square/Fisher's exact test. p-value < 0.05 is statistically significant.

Parameters	USG		MRI		P value*
	Frequency	Percentage	Frequency	Percentage	
Details of findings					
Benign	51	76.1	53	79.1	0.33
Malignant	16	23.9	14	20.9	
Size of tumor					
<5 cm	15	22.4	10	14.9	0.5
5-10 cm	32	47.8	36	53.7	
10-15 cm	17	25.4	15	22.4	
>15 cm	3	4.5	6	9.0	
Surface of tumor					
Regular	41	61.2	38	56.7	0.29
Irregular	26	38.8	29	43.3	
Septations					
Present	23	34.3	32	47.8	0.05
Absent	44	65.7	35	52.2	
Septal thickness					
<3 mm	7	4.5	13	8.7	0.21
>3 mm	16	10.7	19	12.7	
Ascites					
Present	9	13.5	10	15	0.4
Absent	58	86.5	57	85	
Lymphadenopathy					
Present	-	-	4	6	-
Absent	-	-	63	94	
Areas of solid components					
Present	23	34.3	32	47.8	0.05
Absent	44	65.7	35	52.2	
Vascularity of the tumor mass					
Increased	22	32.8	-	-	-
Normal	45	67.2	-	-	-

Diagnostic performance

The diagnostic performance of imaging findings with histopathology is demonstrated in Table 5. For USG, 50 masses identified as benign were confirmed benign on histopathology, while 12 of 16 masses classified as malignant were confirmed malignant, yielding a sensitivity of 92.59%, specificity of 92.31%, positive predictive value (PPV) of 98.04%, negative predictive value (NPV) of 75.00%, and an overall diagnostic accuracy of 92.54%. MRI showed slightly superior performance, correctly identifying all 53 benign lesions and 13 of 14 malignant lesions, with a sensitivity of 98.15%, specificity of 100%, PPV of 100%, NPV of 92.86%, and an overall diagnostic accuracy of 98.51%. When USG findings were compared with MRI, 50 benign and 13 malignant masses were concordant, with a sensitivity of 94.34%, specificity of 92.86%, PPV of 98.04%, NPV of 81.25%, and an overall agreement of 94.03%.

**Table 5:** Diagnostic performance of USG, MRI, and HPE among the study participants (n = 67) This table shows the Diagnostic performance of USG, MRI, and HPE among the study participants, expressed as sensitivity, specificity, and positive and negative predictive values. USG: ultrasonography, HPE: histopathological examination, MRI: magnetic resonance imaging.

Type	Scan Findings	Benign	Malignant	Sensitivity (%)	Specificity (%)	Positive Predictive Value (%)	Negative Predictive Value (%)	Diagnostic Accuracy (%)
USG vs HPE	Benign	50	1	92.59	92.31	98.04	75.00	92.54
	Malignant	4	12					
MRI vs HPE	Benign	53	0	98.15	100	100	92.86	98.51
	Malignant	1	13					
USG vs MRI	Benign	50	1	94.34	92.86	98.04	81.25	94.03
	Malignant	3	13					

## Discussion

This study evaluated the clinical, imaging, and histopathological characteristics of patients with adnexal masses. Most patients were middle-aged or older, predominantly in the 30-40 and 50-60-year age groups, consistent with prior studies showing that adnexal masses are more common in women of reproductive and perimenopausal age [13-17]. The small proportion of patients above 60 years reflects the lower prevalence in elderly women, while younger age groups were less affected.

Clinically, lower abdominal pain was the most frequent presenting symptom, followed by pelvic heaviness and urinary complaints. A palpable mass was rare, highlighting that pain is the predominant symptom in adnexal pathology [16,17]. Similar observations were reported by Nitya et al. and Siddhartha et al. [15,16], where abdominal pain was the major complaint, while other symptoms such as backache, amenorrhea, and abdominal distension were uncommon. Aswini et al. also reported lower abdominal pain and palpable mass as primary clinical features, supporting the importance of symptom-driven imaging evaluation [17].

Obstetric history showed that most patients were parous, with a minority being nulliparous. The majority had no history of cesarean section, suggesting that previous surgical deliveries may not significantly contribute to adnexal mass development. Menopausal status was nearly evenly distributed, though slightly more patients were premenopausal, consistent with prior studies [13]. These findings emphasize that adnexal masses occur across reproductive and postmenopausal ages, necessitating imaging evaluation in both groups.

USG effectively classified the majority of adnexal lesions as benign, corroborating its role as a first-line diagnostic modality [14-16]. USG also provided key morphological information such as tumor surface regularity, septations, and solid components, which aid in malignancy risk assessment. Increased vascularity observed on color Doppler can indicate potential malignant transformation, underscoring the importance of comprehensive USG assessment [14].

MRI demonstrated slightly higher detection rates of benign lesions and superior characterization of tumor morphology, solid components, septations, and peritoneal fluid, highlighting its value in complex or indeterminate cases [18,19]. MRI’s ability to detect ascites and lymphadenopathy more reliably than USG reflects its higher sensitivity and improved tissue resolution, which is critical for preoperative planning and staging. Tumor surface evaluation and septal thickness assessment on MRI further enhance differentiation between benign and suspicious lesions, supporting MRI as a complementary tool to USG in adnexal mass evaluation.

Histopathology confirmed that benign cystic lesions were the most common adnexal masses, including simple cysts, serous cystadenomas, hydrosalpinx, and mucinous cystadenomas, while malignant lesions were relatively rare [13-15]. This aligns with previously published data indicating a predominance of benign lesions in adnexal pathology. Imaging findings correlated well with histopathological diagnoses, with USG demonstrating high sensitivity and specificity, while MRI provided near-perfect diagnostic performance [16,17]. These observations underscore the complementary roles of USG and MRI, where USG serves as a cost-effective screening tool and MRI enhances diagnostic confidence, particularly for complex or suspicious masses.

Correlation between USG and MRI findings showed high agreement, reflecting that USG is a reliable initial modality while MRI can refine lesion characterization. Prior studies support this approach, showing that USG is highly effective in identifying malignant lesions, although MRI provides improved sensitivity and specificity [14-17]. Notably, USG may occasionally miss complex or small malignant lesions, highlighting the need for MRI when features are indeterminate or suggestive of malignancy.

The study emphasizes several important imaging features that guide clinical management. Tumor morphology, solid components, septations, vascularity, and the presence of ascites or lymphadenopathy are crucial in distinguishing benign from malignant masses. MRI’s multiplanar capability and superior soft tissue contrast enhance lesion characterization, staging, and surgical planning. Early and accurate differentiation between benign and malignant adnexal masses can prevent unnecessary radical surgery in benign cases and ensure timely intervention in malignancies, optimizing patient outcomes [20].

This study has some limitations. The sample size was relatively small, and the study was conducted at a single center, which may limit generalizability. Selection bias may exist, as patients with inconclusive or symptomatic masses were preferentially included. Not all patients underwent surgery, limiting histopathological correlation for every lesion. Additionally, MRI is less accessible and more costly than USG, which may restrict its routine use in resource-limited settings.

Despite these limitations, the study highlights the complementary role of USG and MRI in the evaluation of adnexal masses. USG remains an effective, accessible first-line modality, while MRI significantly enhances diagnostic confidence for complex or suspicious lesions. Recognition of specific imaging features associated with malignancy can guide clinical decision-making, optimize surgical planning, and reduce morbidity. Future studies with larger, multicenter cohorts are warranted to validate these findings and refine imaging protocols for better risk stratification and patient management.

## Conclusions

In this study, USG proved to be a reliable first-line imaging modality for evaluating adnexal masses, effectively distinguishing benign from malignant lesions with high sensitivity and specificity. MRI demonstrated superior diagnostic performance, particularly in characterizing complex masses and detecting solid components, septations, ascites, and lymphadenopathy, thereby enhancing preoperative assessment and clinical decision-making. Histopathology confirmed that benign cystic lesions were predominant, while malignancies were relatively uncommon. The combined use of USG and MRI allows accurate diagnosis, optimal surgical planning, and improved patient outcomes, underscoring the importance of a multimodal imaging approach in the evaluation of adnexal masses.
